# Bioinformatics Curriculum Guidelines: Toward a Definition of Core Competencies

**DOI:** 10.1371/journal.pcbi.1003496

**Published:** 2014-03-06

**Authors:** Lonnie Welch, Fran Lewitter, Russell Schwartz, Cath Brooksbank, Predrag Radivojac, Bruno Gaeta, Maria Victoria Schneider

**Affiliations:** 1 School of Electrical Engineering and Computer Science, Ohio University, Athens, Ohio, United States of America; 2 Bioinformatics and Research Computing, Whitehead Institute, Cambridge, Massachusetts, United States of America; 3 Department of Biological Sciences and School of Computer Science, Carnegie Mellon University, Pittsburgh, Pennsylvania, United States of America; 4 European Molecular Biology Laboratory, European Bioinformatics Institute, Wellcome Trust Genome Campus, Hinxton, Cambridge, United Kingdom; 5 School of Informatics and Computing, Indiana University, Bloomington, Indiana, United States of America; 6 School of Computer Science and Engineering, The University of New South Wales, Sydney, New South Wales, Australia; 7 The Genome Analysis Centre, Norwich Research Park, Norwich, United Kingdom

## Introduction

Rapid advances in the life sciences and in related information technologies necessitate the ongoing refinement of bioinformatics educational programs in order to maintain their relevance. As the discipline of bioinformatics and computational biology expands and matures, it is important to characterize the elements that contribute to the success of professionals in this field. These individuals work in a wide variety of settings, including bioinformatics core facilities, biological and medical research laboratories, software development organizations, pharmaceutical and instrument development companies, and institutions that provide education, service, and training. In response to this need, the Curriculum Task Force of the International Society for Computational Biology (ISCB) Education Committee seeks to define curricular guidelines for those who train and educate bioinformaticians. The previous report of the task force summarized a survey that was conducted to gather input regarding the skill set needed by bioinformaticians [1]. The current article details a subsequent effort, wherein the task force broadened its perspectives by examining bioinformatics career opportunities, surveying directors of bioinformatics core facilities, and reviewing bioinformatics education programs.

The bioinformatics literature provides valuable perspectives on bioinformatics education by defining skill sets needed by bioinformaticians, presenting approaches for providing informatics training to biologists, and discussing the roles of bioinformatics core facilities in training and education.

The skill sets required for success in the field of bioinformatics are considered by several authors: Altman [2] defines five broad areas of competency and lists key technologies; Ranganathan [3] presents highlights from the Workshops on Education in Bioinformatics, discussing challenges and possible solutions; Yale's interdepartmental PhD program in computational biology and bioinformatics is described in [4], which lists the general areas of knowledge of bioinformatics; in a related article, a graduate of Yale's PhD program reflects on the skills needed by a bioinformatician [5]; Altman and Klein [6] describe the Stanford Biomedical Informatics (BMI) Training Program, presenting observed trends among BMI students; the American Medical Informatics Association defines competencies in the related field of biomedical informatics in [7]; and the approaches used in several German universities to implement bioinformatics education are described in [8].

Several approaches to providing bioinformatics training for biologists are described in the literature. Tan et al. [9] report on workshops conducted to identify a minimum skill set for biologists to be able to address the informatics challenges of the “-omics” era. They define a requisite skill set by analyzing responses to questions about the knowledge, skills, and abilities that biologists should possess. The authors in [10] present examples of strategies and methods for incorporating bioinformatics content into undergraduate life sciences curricula. Pevzner and Shamir [11] propose that undergraduate biology curricula should contain an additional course, “Algorithmic, Mathematical, and Statistical Concepts in Biology.” Wingren and Botstein [12] present a graduate course in quantitative biology that is based on original, pathbreaking papers in diverse areas of biology. Johnson and Friedman [13] evaluate the effectiveness of incorporating biological informatics into a clinical informatics program. The results reported are based on interviews of four students and informal assessments of bioinformatics faculty.

The challenges and opportunities relevant to training and education in the context of bioinformatics core facilities are discussed by Lewitter et al. [14]. Relatedly, Lewitter and Rebhan [15] provide guidance regarding the role of a bioinformatics core facility in hiring biologists and in furthering their education in bioinformatics. Richter and Sexton [16] describe a need for highly trained bioinformaticians in core facilities and provide a list of requisite skills. Similarly, Kallioniemi et al. [17] highlight the roles of bioinformatics core units in education and training.

This manuscript expands the body of knowledge pertaining to bioinformatics curriculum guidelines by presenting the results from a broad set of surveys (of core facility directors, of career opportunities, and of existing curricula). Although there is some overlap in the findings of the surveys, they are reported separately, in order to avoid masking the unique aspects of each of the perspectives and to demonstrate that the same themes arise, even when different perspectives are considered. The authors derive from their surveys an initial set of core competencies and relate the competencies to three different categories of professions that have a need for bioinformatics training.

## Survey of Directors of Bioinformatics Core Facilities

Bioinformatics educational programs face the risk of producing students who have skills that are primarily academic in nature, thereby limiting the utility of program graduates. To investigate this risk, the ISCB Curriculum Task Force sought to capture the perspectives of directors of bioinformatics core facilities as representatives of employers of professional bioinformaticians. Specifically, the core facility directors were asked what skills are needed for success in the field of bioinformatics and what skills are lacking in recently hired bioinformaticians. In general, these lists were very similar (i.e., skills needed are often lacking). Twenty-nine core facility directors responded to the survey. The respondents were from Europe (six), Israel (one), and the United States and Canada (21). (One respondent did not indicate geographic location.) The results are divided into general skills and domain-specific skills and are categorized by level of training: bachelors (ten respondents), masters (22 respondents), and PhDs (25 respondents).

Hiring at the *bachelor level* appears to be a less frequent occurrence than hiring people with graduate degrees. At the bachelor level, managers are looking for people who can work independently, have good communications and consulting skills, are organized, and are passionate about their work. The most frequently mentioned domain-specific skills needed for bachelor-level candidates were technical in nature and included programming, software engineering, system administration, and databases. New hires for such positions at the bachelor level typically lack time management skills and project management skills and are unable to manage multiple projects. They also lack knowledge in biology and statistics.

The responses for hiring at the *master level* were far more numerous and varied. General skills needed include those that are more interpretative and problem solving, as well as personal traits, such as being independent, curious, and self-motivated. These same skills are considered lacking in many master-level hires. With respect to domain-specific skills, directors need people well versed in biology, bioinformatics, statistics, and programming, essentially needing people with technical experience in both biological sciences and computational methods. New hires often lack experience in the analysis of real biological data.

Not surprisingly, general skills needed at the PhD level include those skills necessary at the master level, as well as communications skills, management skills, and the ability to help others. Skills most frequently found lacking in individuals with PhDs include communications skills, ability to synthesize information, ability to complete projects, and leadership skills. The domain-specific skills were similar to those needed at the master level, but emphasized more prior experience in bioinformatics, data analysis, and statistics. What is lacking among candidates at this level is experience specific to work done by the hiring group.

The responses of the core facility directors can be summarized as follows: everyone wants smart, motivated people with good critical thinking skills and deep domain knowledge. It is clear that training in both general skills and domain-specific skills is necessary at all professional levels, both while in a degree program and throughout one's career. Table 1 presents the skill sets synthesized from the bioinformatics core facility directors' survey and the bioinformatics career opportunity survey.

**Table 1:** Summary of the skill sets of a bioinformatician, identified by surveying bioinformatics core facility directors and examining bioinformatics career opportunities.

Skill Category	Specific Skills
General	time management, project management, management of multiple projects, independence, curiosity, self-motivation, ability to synthesize information, ability to complete projects, leadership, critical thinking, dedication, ability to communicate scientific concepts, analytical reasoning, scientific creativity, collaborative ability
Computational	programming, software engineering, system administration, algorithm design and analysis, machine learning, data mining, database design and management, scripting languages, ability to use scientific and statistical analysis software packages, open source software repositories, distributed and high-performance computing, networking, web authoring tools, web-based user interface implementation technologies, version control tools
Biology	molecular biology, genomics, genetics, cell biology, biochemistry, evolutionary theory, regulatory genomics, systems biology, next generation sequencing, proteomics/mass spectrometry, specialized knowledge in one or more domains
Statistics and Mathematics	application of statistics in the contexts of molecular biology and genomics, mastery of relevant statistical and mathematical modeling methods (including experimental design, descriptive and inferential statistics, probability theory, differential equations and parameter estimation, graph theory, epidemiological data analysis, analysis of next generation sequencing data using R and Bioconductor)
Bioinformatics	analysis of biological data; working in a production environment managing scientific data; modeling and warehousing of biological data; using and building ontologies; retrieving and manipulating data from public repositories; ability to manage, interpret, and analyze large data sets; broad knowledge of bioinformatics analysis methodologies; familiarity with functional genetic and genomic data; expertise in common bioinformatics software packages, tools, and algorithms

## Survey of Career Opportunities

The context in which bioinformaticians employ their talents is an important consideration for defining bioinformatics curricular guidelines. Thus, we analyzed the *ISCB - Membership Job Board* postings (see http://www.iscb.org/iscb-careers) to determine the responsibilities and required skills of bioinformaticians. We examined job listings from a four-month period, sampling 75 listings (of 130) from diverse geographic locations. Specifically, job listings from the following locations were analyzed: Australia, Austria, Canada (London, Ottawa, Toronto), China (Hong Kong, Shanghai), Denmark, France, Germany, Israel, Italy, Japan, Kenya, Singapore, South Africa, South Korea, Sweden (Stockholm, Uppsala), the United Kingdom (Cambridge, London, Norwich), and the United States (Arizona, Georgia, Texas, Delaware, North Carolina, California, Colorado, Iowa, Illinois, Indiana, Kansas, Massachusetts, Maryland, New York, Pennsylvania, Michigan). The remainder of this section summarizes the duties and skills required for the bioinformatics positions considered.

The responsibilities of a bioinformatician include data analysis, software development, project support, and computational infrastructure support in biological contexts (such as next generation sequencing, medical research, regulatory genomics, and systems biology).

A bioinformatician analyzes and manages data as a member of an interdisciplinary research team composed of members from disciplines that span the biological, medical, computational, and mathematical sciences. This involves several activities: working in a production environment managing scientific data; modeling, building, and warehousing biological data; using and/or building ontologies; and retrieving, manipulating, and managing data from public data repositories.

To successfully perform the duties of a bioinformatician, one must possess an array of bioinformatics skills: ability to manage, interpret, and analyze large data sets; broad knowledge of bioinformatics analysis methodologies; familiarity with functional genetic and genomic data; and expertise in common bioinformatics software packages and algorithms.

A bioinformatician must apply statistics in contexts such as molecular biology, genomics, and population genetics. Thus, a bioinformatician must have mastery of relevant statistical and mathematical modeling methods, including descriptive and inferential statistics, probability theory, differential equations and parameter estimation, graph theory, epidemiological data analysis, and programming and analysis of next generation sequencing data using software such as R and Bioconductor.

The ability to employ computer science methods is critical in the discipline of bioinformatics because custom software tools and databases often need to be created. Therefore, a bioinformatician must have the ability to apply software engineering methodologies to successfully design, implement, and maintain systems and software in scientific environments. The ability to employ modern software engineering processes (such as object-oriented analysis, design, and implementation) is important. In order to develop efficient and effective software systems, it is valuable to have a detailed understanding of the methods of algorithm design and analysis, machine learning, data mining, and relational databases. A bioinformatician should be proficient in the use of one or more scripting languages (such as Perl, Python, Java, C, C++, C#, .NET, and Ruby), database management languages (e.g., Oracle, PostgreSQL, and MySQL), and scientific and statistical analysis software (such as R, S-plus, MATLAB, and Mathematica). Additionally, a bioinformatician should be able to incorporate components from open source software repositories into a software system. The ability to effectively utilize distributed and high-performance computing to analyze large data sets is essential, as is knowledge of networking technology and internet protocols. A bioinformatician should be able to utilize web authoring tools, web-based user interface implementation technologies, and version control and build tools (e.g., subversion, Ant, and Netbeans).

While it is important for a bioinformatician to have a suite of computational, mathematical, and statistical skills, this alone is insufficient. Throughout their careers, bioinformaticians usually contribute to a variety of scientific projects, such as variant detection in human exome resequencing; human genetic diversity; genomic and epigenomic mechanisms of gene regulation; viral diversity; neurodegeneration and psychiatric disorders; drug discovery; the role of transcription factors and chromatin structure in global gene expression, development, and differentiation; and cancer/tumor biology. To be a fully integrated member of a research team, a bioinformatician must possess detailed knowledge of molecular biology, genomics, genetics, cell biology, biochemistry, and evolutionary theory. Furthermore, it is necessary to understand related technologies, including next generation sequencing and proteomics/mass spectrometry. It is also desirable for a bioinformatician to have modeling experience or background in one or more specialized domains, such as systems biology, inflammation, immunology, cell signaling, or physiology.

Additionally, a bioinformatician must have a high level of motivation, be independent and dedicated, possess strong interpersonal and managerial skills, and have outstanding analytical ability. A bioinformatician must have excellent teamwork skills and have strong scientific communication skills.

As a bioinformatician progresses through his or her career, it is helpful to develop managerial and programmatic skills, such as staff management and business development; understanding of or experience with grant funding and/or access to finance; awareness of research and development (R&D) and innovation policy and government drivers; the use of modeling and simulation approaches; ability to evaluate the major factors associated with efficacy and safety; and ability to answer regulatory questions related to product approval and risk management. It is also important to have familiarity with presenting biological results in both oral and written forms.

In summary, a senior bioinformatician will benefit from strong analytical reasoning capabilities, as evidenced by a track record of innovation; scientific creativity, collaborative ability, mentoring skills, and independent thought; and a record of outstanding research. Table 1 summarizes the skill sets identified by (1) surveying bioinformatics core facility directors and (2) examining bioinformatics career opportunities.

## Preliminary Survey of Existing Curricula

An important step in developing guidelines for bioinformatics education is to gain a comprehensive understanding of current practices in bioinformatics and computational biology education. To this end, the task force surveyed and catalogued existing curricula used in bioinformatics educational programs.

As a first step, the task force began a manual search for educational programs. Due to the large number of education programs, the decision was made to initially restrict the search to programs awarding a degree or certificate and explicitly including “computational biology,” “bioinformatics,” or some close variant in the name of the degree or certificate awarded. The search thus excluded non-degree tracks or options within more traditional programs, non-degree programs of study, or programs in related fields that might have high overlap with bioinformatics (e.g., biostatistics or biomedical informatics). Although this was a controversial decision even within the task force, this narrow scope and definition of programs was intended to keep the search from becoming too unfocused or being sidetracked over questions of which programs should be included as belonging to the field.

A search by committee members produced a preliminary collection of two programs awarding degrees of associate of arts or sciences; 72 awarding bachelor of science, arts, or technology; 38 awarding master of science, research, or biotechnology; 39 awarding doctor of philosophy; and 15 awarding non-degree certificates. However, it provided a basis for manual examination of trends in educational practice.

Attempts to identify common practices among this narrow subset revealed substantial challenges. First, differences in types of degrees and regulations for awarding them proved challenging in making a precise but inclusive definition of a bioinformatics degree program, especially across international boundaries. Differences in how specific topics are partitioned among courses and limited information on the contents of specific courses likewise hindered analysis. For example, multiple programs may have a class called “Bioinformatics I,” yet one cannot assume these classes cover comparable material. Furthermore, the number of extant programs and the lack of any central repository of information or standard reporting format make it difficult to make any comprehensive statements about current accepted practices or variations. Finally, the preliminary surveys revealed an extraordinary diversity of requirements across programs, even at a given degree level. Consequently, it was extremely difficult to catalog the requirements for an individual program and a greater challenge to identify the commonalities between programs.

Given the challenges of conducting a committee-directed survey, the task force concluded that self-reporting of program features by cognizant program officials would be the best mechanism to produce a survey that is comprehensive, inclusive, and accurate. The task force hopes to have, in the future, a central system in which program officials can identify their programs and describe the coursework they require, yielding a database that can be mined to uncover common practices and variations across programs at multiple levels. Such a repository could be made available for public viewing, as we expect it will have incidental benefits for others, such as potential students looking to compare programs.

A key obstacle to creating such a repository has been identifying a format that allows the coursework to be categorized in a way that is specific enough to meaningfully distinguish among programs but general enough to allow one to identify commonalities among classes that are never identical across institutions. To this end, a decision was made to produce a controlled vocabulary in which programs can report their required courses.

**Figure 1:**
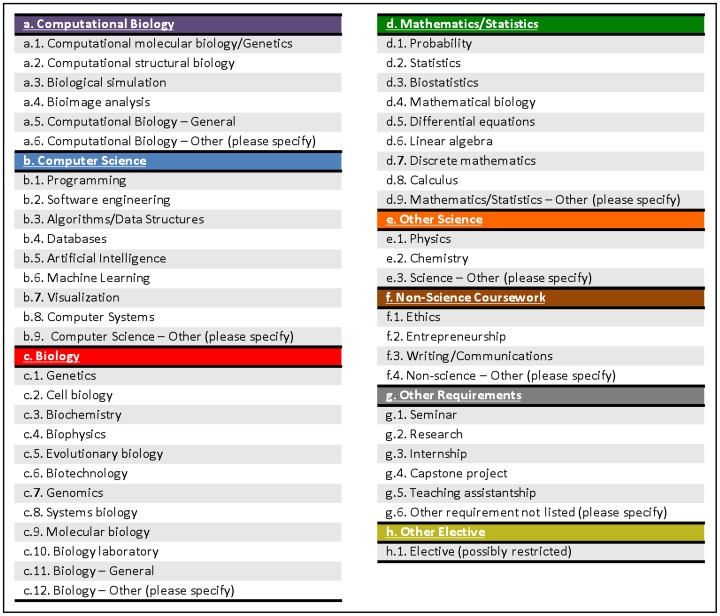
Draft of a controlled vocabulary for identifying specific requirements of computational biology and bioinformatics degree and certificate programs. The terms are drawn from requirements observed in a manual survey of a subset of existing educational programs in order to allow identification of recurring requirements while also allowing for the wide variation between programs.

Figure 1 provides an initial draft of such a controlled vocabulary, which was developed manually, based on the initial task force survey of existing curricula. We note that this is not intended to be a finished product but rather a starting point for discussion. We hope for feedback, to improve this vocabulary in order to represent the range of variation in classes offered by such programs.

The task force intends to incorporate the final controlled vocabulary into a website to which individual program officials can add their programs, providing identifying information and a description of the curriculum in terms of the vocabulary. This is a task that will require community participation, and it is our hope that a shared desire to identify best practices and the benefits of having a program listed in a central repository will encourage broad participation.

## Discussion

### Toward a definition of core competencies

In the discipline of bioinformatics and computational biology, there are numerous ways in which curricula can be designed to achieve the desired educational outcomes. However, analysis of our survey results suggests that there is a common set of desired proficiencies for bioinformaticians. We have organized these desired proficiencies into a set of core competencies to provide guidance for bioinformatics educational programs. These guidelines synthesize the results of our surveys (see preceding sections of this manuscript). While we acknowledge that we are dealing with small samples of responses, not randomly surveyed, the resulting competencies do not contravene previously published recommendations (see introduction and references [1]–[16]), and they comport with the experiences of the task force members. The wording for the core competencies is modeled after the Accreditation Board for Engineering and Technology (ABET) criteria for computer science programs [18], using the terminology and concepts of Bloom's Taxonomy [19]–[21]. Our recommendation is that bioinformatics programs enable students to attain the competencies shown in the rows of Table 2.

**Table 2:** Core competencies for each bioinformatics training category.

	Bioinformatics User	Bioinformatics Scientist	Bioinformatics Engineer
(a) An ability to apply knowledge of computing, biology, statistics, and mathematics appropriate to the discipline.		X	X
(b) An ability to analyze a problem and identify and define the computing requirements appropriate to its solution.		X	X
(c) An ability to design, implement, and evaluate a computer-based system, process, component, or program to meet desired needs in scientific environments.			X
(d) An ability to use current techniques, skills, and tools necessary for computational biology practice.	X	X	X
(e) An ability to apply mathematical foundations, algorithmic principles, and computer science theory in the modeling and design of computer-based systems in a way that demonstrates comprehension of the tradeoffs involved in design choices.			X
(f) An ability to apply design and development principles in the construction of software systems of varying complexity.			X
(g) An ability to function effectively on teams to accomplish a common goal.	X	X	X
(h) An understanding of professional, ethical, legal, security, and social issues and responsibilities.	X	X	X
(i) An ability to communicate effectively with a range of audiences.	X	X	X
(j) An ability to analyze the local and global impact of bioinformatics and genomics on individuals, organizations, and society.	X	X	X
(k) Recognition of the need for and an ability to engage in continuing professional development.	X	X	X
(l) Detailed understanding of the scientific discovery process and of the role of bioinformatics in it.	X	X	X
(m) An ability to apply statistical research methods in the contexts of molecular biology, genomics, medical, and population genetics research.	X	X	X
(n) Knowledge of general biology, in-depth knowledge of at least one area of biology, and understanding of biological data generation technologies.	X	X	X

It is not the intention of the authors to imply that the skill set of one category is entirely subsumed by the skill set of another category. The focus of this document is on bioinformatics; thus, the authors did not attempt to define the full set of competencies that are required in the medical, legal, and scientific contexts.

The columns of Table 2 indicate core competencies for three different types of individuals that have a need for bioinformatics training. (The three categories of bioinformatics training are not meant to capture all possible types of bioinformatics training needed but to describe three common categories.) *Bioinformatics users* access data resources to perform job duties in specific application domains. Bench-based researchers, both in academia and in industry, provide the classic example of a bioinformatics user, but this group is broadening in scope. For example, medical professionals (e.g., physicians and genetic counselors) utilize bioinformatics resources in medical contexts for the purposes of diagnosis, treatment, and counseling of patients. As the practices of genomic and personalized medicine increase, we anticipate a growing need for training clinicians in the use of bioinformatics data and tools. Other bioinformatics users include legal professionals and K-12 biology teachers.

The authors use personas to refine their understanding of different types of computational biologists and the competencies that they require to perform their roles. Designers of commercial products frequently create “personas”—archetypes based on data and research on the users for whom a product is being designed—to facilitate the design process. This technique is beginning to pervade the design of bioinformatics resources [22], [23]. The use of personas can also be extremely powerful in educational contexts. Personas have two important functions. First, they can help to guide decisions about the appropriateness of the course or curriculum under development: we can ask questions such as, “How might the removal of module A affect the workflow of trainee B?” Second, they can create empathy, reminding the course developer (and ultimately the trainer) that the trainee might have different end goals than her/his own.

**Figure 2:**
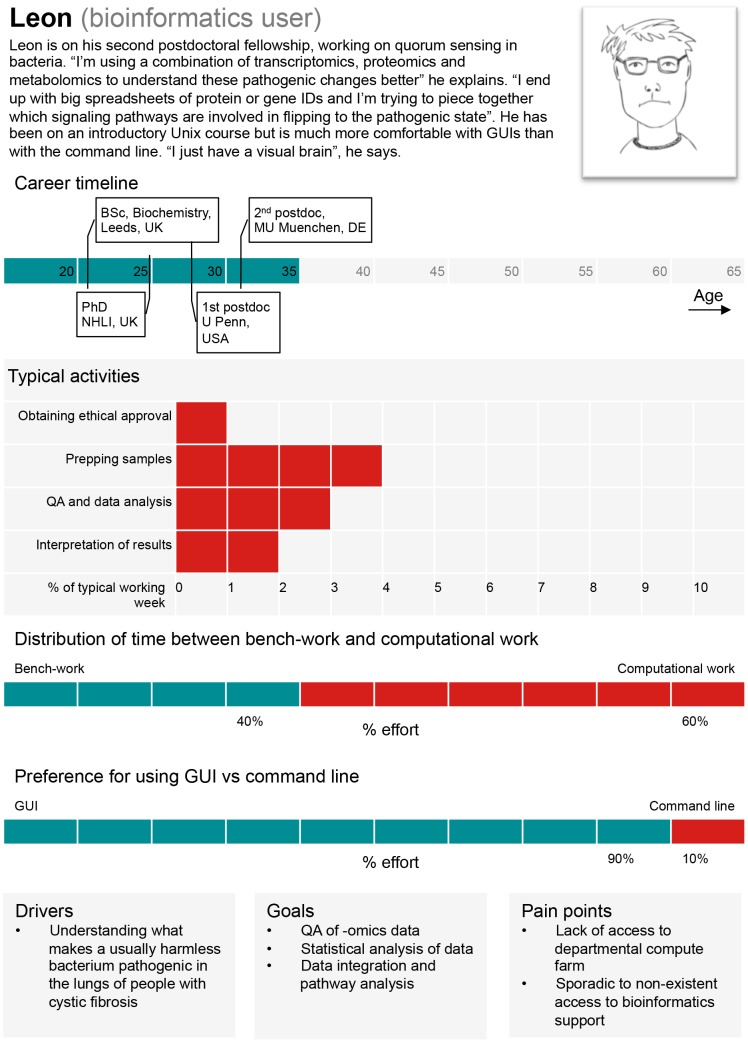
A persona based on a typical “bioinformatics user.” QA: Quality Assurance, GUI: Graphical User Interface. Image credit: Jenny Cham, Mary Todd Bergman, and Cath Brooksbank, EMBL-EBI.

An example persona for a bioinformatics user is provided in Figure 2. This persona, based on a typical “bioinformatics user,” can help a curriculum designer to interpret the core competencies in Table 2. For example, in training for the competency, “(d) An ability to use current techniques, skills, and tools necessary for computing practice,” one should consider including adequate time for familiarization with the command line, data management practice, and statistical analysis tools.

**Figure 3:**
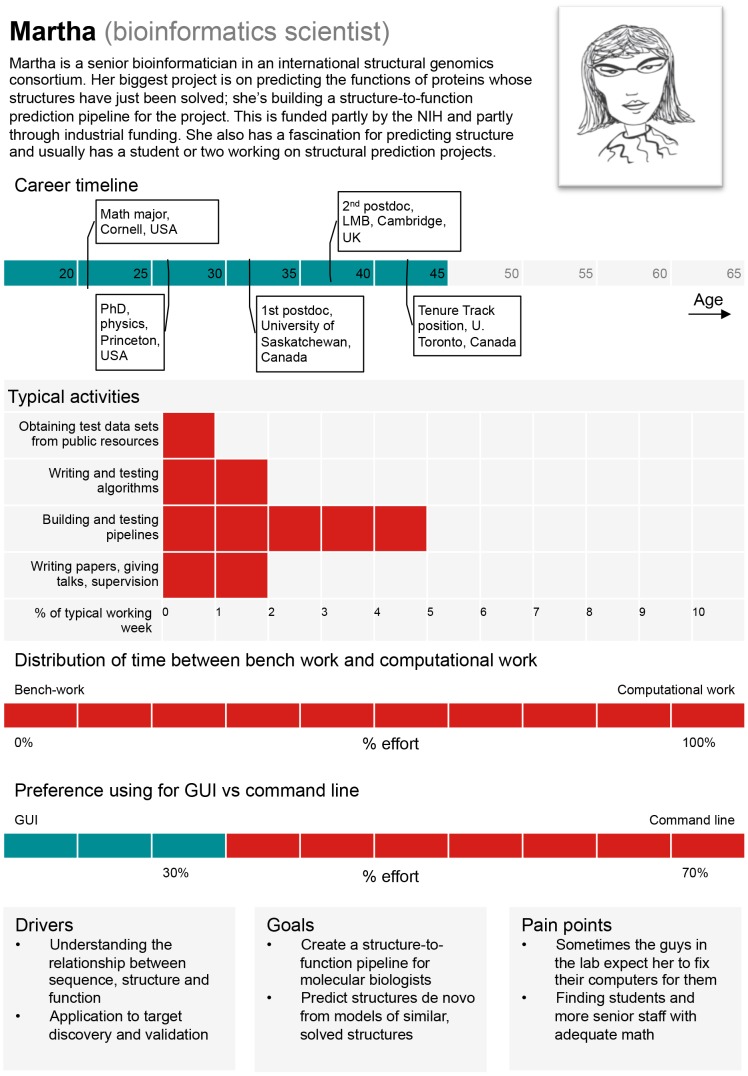
A persona based on a typical “bioinformatics scientist.” GUI: Graphical User Interface. Image credit: Jenny Cham, Mary Todd Bergman, and Cath Brooksbank, EMBL-EBI.

*Bioinformatics scientists* are biologists who employ computational methods in order to advance the scientific understanding of living systems. Both bioinformatics users and bioinformatics scientists should have a basic understanding of the nature of the computational tools they employ, especially when making conclusions based on statistical inference. For example, the *E-value* output of the BLAST software [24] depends on the sequence statistics of the database against which a search is conducted. As many uses of BLAST require search of customized databases, different searches can lead to difficulties in result interpretation and comparison. Thus, basic knowledge of modeling assumptions and how methods were “trained” is critical. A persona for an archetypal “bioinformatics scientist” is provided in Figure 3.

**Figure 4:**
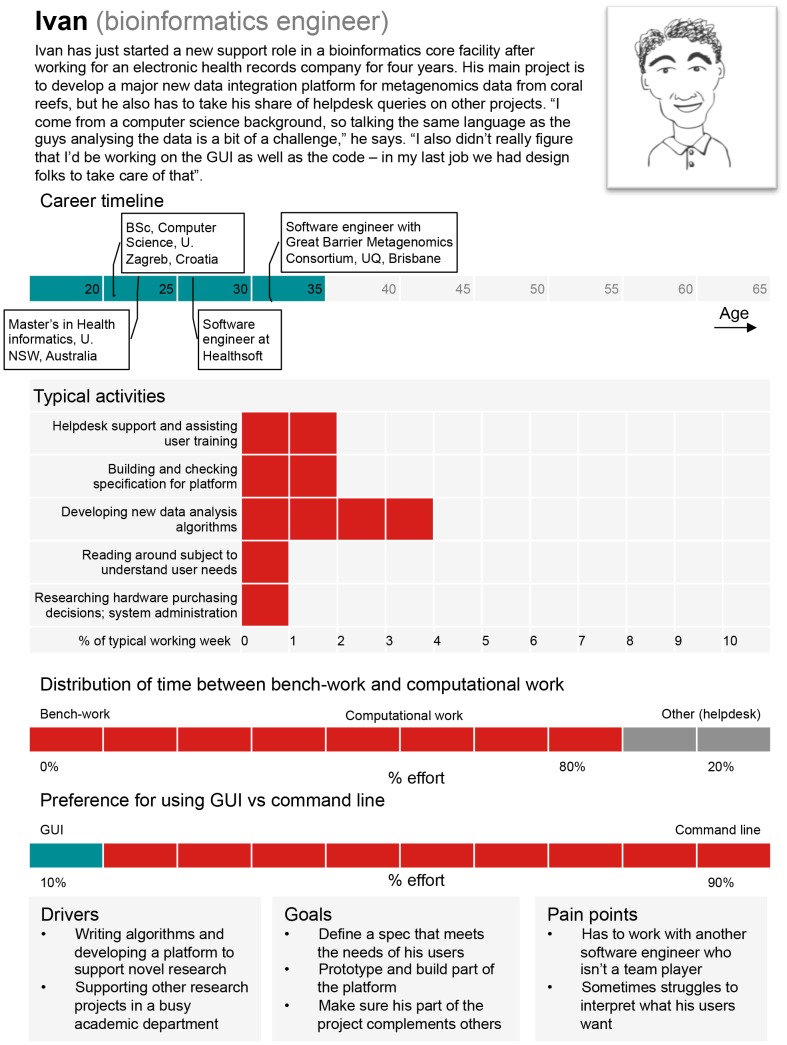
A persona based on a typical “bioinformatics engineer.” GUI: Graphical User Interface. Image credit: Jenny Cham, Mary Todd Bergman, and Cath Brooksbank, EMBL-EBI.

*Bioinformatics engineers* create the novel computational methods needed by bioinformatics users and scientists [25], [26]. Thus, a bioinformatics engineer must have strengths in computational and statistical sciences and must have general competency in biomedical sciences. Bioinformatics engineers design the infrastructure and systems for bioinformatics analysis, integrating software, databases, and hardware. This can involve the choice or design of hardware and software for the storage and management of diverse and distributed data, selection or development of tools and algorithms for integration and analysis of these data, and design of suitable user interfaces. The critical and complex nature of bioinformatics software and the growing volume of associated data require the development of reliable and maintainable systems in an environment where requirements can be complex, vague, and volatile, and budgets and schedules are often tight. In addition to strong scientific foundations and technical skills the bioinformatics engineer needs to bring to bear engineering competencies such as systems design and project management to ensure the quality, viability, and sustainability of the software systems developed. A persona for a representative “bioinformatics engineer” is provided in Figure 4.

### Conclusions

ISCB's Education Committee Curriculum Task Force considered bioinformatics and computational biology training and education in a variety of contexts, resulting in the definition of a broad set of core competencies for three different types of individuals. We hope the concepts presented in the article will be valuable for trainers and educators who wish to design courses and curricula to meet the needs of today's bioinformaticians. The task force will continue to refine and update the curricular guidelines as a service to the bioinformatics community.
